# Pathogenic Drivers of Difficult-to-Treat Rheumatoid Arthritis: Synovium and Beyond

**DOI:** 10.3390/ijms27041860

**Published:** 2026-02-15

**Authors:** András Miklós Dorgó, Lilla Gunkl-Tóth, György Nagy

**Affiliations:** 1 Department of Rheumatology and Immunology, Semmelweis University, 1023 Budapest, Hungary; dorgo.andras.miklos@semmelweis.hu (A.M.D.); gunkl-toth.lilla@semmelweis.hu (L.G.-T.); 2 Department of Pharmacology and Pharmacotherapy, University of Pécs, 7624 Pécs, Hungary; 3 (HUN-REN-PTE) Chronic Pain Research Group, Hungarian Research Network, University of Pécs, 7624 Pécs, Hungary; 4 Heart and Vascular Center, Semmelweis University, 1122 Budapest, Hungary; 5 Department of Genetics, Cell- and Immunobiology, Semmelweis University, 1089 Budapest, Hungary

**Keywords:** rheumatoid arthritis, difficult-to-treat, D2T, treatment failure, inefficacy, b/tsDMARDs, pathogenesis, pain, comorbidity

## Abstract

Difficult-to-treat (D2T) rheumatoid arthritis (RA) remains a major clinical challenge, affecting a significant proportion of patients who experience persistent symptoms despite multiple therapeutic regimens. This narrative review provides a comprehensive overview of potential molecular and cellular mechanisms that may underlie the D2T RA phenotype. We synthesize evidence across a broad biological landscape—the role of genetic and epigenetic factors, autoantibodies, and diverse immune cell subsets is detailed in the context of therapeutic resistance. Furthermore, we examine the potential role of synovial signatures and stromal cell-mediated pathways, which may drive chronicity independently of traditional immune targets. The review highlights the complex interplay of peripheral and central determinants that contribute to patient-reported outcomes such as pain. We also discuss comorbid conditions, environmental factors such as smoking and nutrition, and treatment-related factors relevant to the D2T population. By integrating these aspects, this work aims to facilitate better stratification and the identification of novel therapeutic targets for refractory disease.

## 1. Introduction

Rheumatoid arthritis (RA) is a chronic inflammatory condition estimated to affect about 0.5% of the adult population globally, with women being two to three times more likely to develop the disease than men [1,2]. It typically presents as a symmetrical polyarthritis, predominantly involving the small joints of the hands and feet. Besides musculoskeletal symptoms, systemic complications may appear in the lungs, heart, nervous system, and other organs [3].

Although there is no definitive cure for RA and most patients require lifelong therapy, the adequate use of disease-modifying anti-rheumatic drugs (DMARDs) and glucocorticoids (GCs) can lead to meaningful improvements in disease outcomes. International guidelines highlight the benefits of a treat-to-target (T2T) approach, whereby setting clinical remission—or at least low disease activity—as a key objective guides the shared decision-making process [4]. Conventional synthetic (cs) DMARDs, particularly methotrexate (MTX), remain the cornerstone of initial therapy; however, they fail to sustain adequate long-term symptom relief for many patients. The introduction of biological (b) and targeted synthetic (ts) DMARDs enabled the targeting of specific molecules that are thought to play a role in the pathogenesis of RA, rendering durable disease control a realistic goal in clinical care [5,6]. Furthermore, various non-pharmacological approaches can improve patient-reported outcomes when used alongside drug treatment [7]. Nonetheless, many individuals with RA experience symptoms despite exposure to multiple successive therapeutic regimens, which poses a challenge for both patients and healthcare professionals [8]. Different classes of targeted DMARDs have shown similar efficacy in clinical trials and real-world settings, and evidence-based recommendations do not provide clear-cut guidance on the optimal choice of pharmacotherapy after the failure of conventional drugs. Therefore, the therapeutic trajectory of these individuals is predominantly shaped by safety concerns and financial considerations [9].

RA is a clinically heterogeneous disease: patients exhibit diverse symptoms, disease trajectories, and patterns of joint and systemic involvement, which potentially reflect underlying pathophysiological mechanisms that greatly vary across individuals [10,11]. In light of this, it is expected that the failure of various therapeutic interventions can be explained by a combination of patient-, disease-, and treatment-related factors. Although the prospect of precision medicine, i.e., selection of the best treatment based on the patient’s unique clinical and molecular profile, appears compelling in RA care [12], solid predictive markers with clinical relevance have so far remained elusive [13,14]. In recent years, several large-scale efforts in the high-resolution profiling of synovial tissue [15,16,17] and peripheral blood [18] have advanced our understanding of the biological basis of differential therapeutic responses in RA [19]. Moreover, high-performance computational techniques, particularly machine learning models, are increasingly being developed with the goal of reliably predicting treatment outcomes [20]. Given the apparent complexity of biological determinants, it is still unclear as to what extent these emerging approaches can be integrated into routine clinical settings [21].

Notably, a European Alliance of Associations for Rheumatology (EULAR) task force was established in 2018 to bridge the research and care gaps associated with patients failing multiple therapies. This led to the development of a consensus definition of difficult-to-treat (D2T) RA [22], which was followed by the publication of evidence-based points to consider for its management [23]. This initiative focuses on patients who continue to have active/progressive disease despite exposure to ≥2 targeted treatments with different mechanisms of action. A recent meta-analysis of observational studies following the EULAR definition reported a pooled prevalence of 10.9% for D2T RA across general RA populations [24].

Due to its permissive design, the D2T RA framework covers a heterogeneous patient cohort [25], and various studies have sought to stratify D2T disease, yet no formal subgroups have been established to date. Alongside the consensus definition for D2T RA, various concepts related to RA treatment failure are outlined in the recent literature (Table 1). An important distinction lies in the assessment of the presence or absence of ongoing inflammation [23,26], and it is estimated that about half of patients with D2T RA exhibit evidence of persistent inflammatory activity [24]. Conceptually, ‘true refractory’ RA may occur in a smaller proportion of patients, in which certain immunological factors confer resistance to most (or all) available treatments, potentially leading to persistent inflammation [27]. However, the prevailing mechanisms that account for poor response across treatment lines in this tentative disease subset remain poorly understood.

When inflammation is minimal or absent, subjective symptoms (e.g., pain or fatigue) may shape the clinical picture and can be amplified by comorbid conditions such as osteoarthritis, fibromyalgia (FM), depression, anxiety, obesity, cardiovascular disease (CVD), or chronic lung involvement. Patient-related factors (e.g., adherence, socioeconomic factors) and suboptimal healthcare journeys (e.g., diagnostic delays, limited treatment access) are also critical considerations in D2T RA [28], although largely beyond the scope of this paper. Importantly, some individuals can regain disease control following a period in the D2T state, adding a temporal dimension to the concept [29,30].

**Table 1:** The framework of difficult-to-treat disease and related concepts used in the recent literature.

Term	Meaning	Ref.
**Difficult-to-treat (D2T) RA**	Failure of multiple b/tsDMARD classes (regardless of reason) ^1^ + active/progressive disease + clinical perception as problematic	[22,23]
**Refractory RA**	Inefficacy of multiple DMARDs	[26,31]
*Persistent inflammatory refractory RA (PIRRA)*	Refractory RA + persistent joint (±systemic) inflammation	[26]
*Non-inflammatory refractory RA (NIRRA)*	Refractory RA *without* objective evidence of ongoing inflammation	[26]
**True refractory RA**	Presence of immunological drivers → inefficacy of multiple (or all) DMARDs (±persistent inflammation)	[27,31]
**Poly-refractory RA**	Failure of all available b/tsDMARD classes (due to inefficacy or intolerance)	[32]

^1^ The current EULAR definition designates failure of ≥2 b/tsDMARD classes as the threshold for classifying D2T RA. Abbreviations: b, biological; D2T, difficult-to-treat; DMARD, disease-modifying anti-rheumatic drug; NIRRA, non-inflammatory refractory rheumatoid arthritis; PIRRA, persistent inflammatory refractory rheumatoid arthritis; RA, rheumatoid arthritis; ts, targeted synthetic.

**Figure 1:**
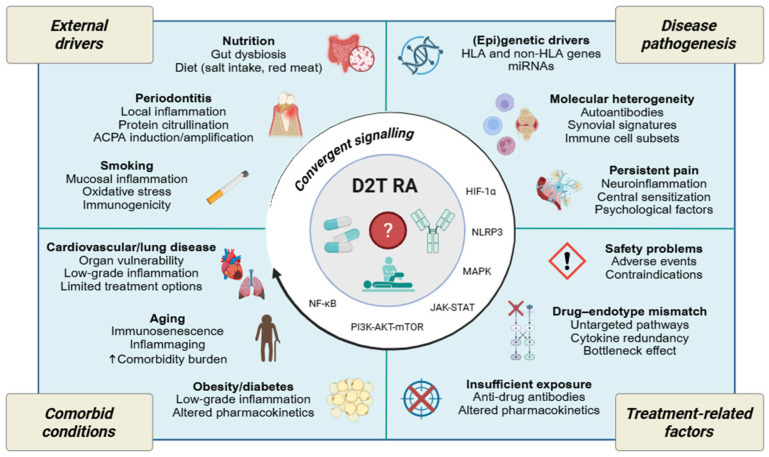
Overview of potential pathogenic mechanisms and contextual factors contributing to difficult-to-treat (D2T) rheumatoid arthritis (RA). External drivers (e.g., smoking, periodontitis, gut dysbiosis, diet) may promote mucosal and systemic immune activation. Comorbidities (obesity/diabetes, cardiovascular disease, interstitial lung disease, and age-related changes) can add a low-grade inflammatory background, influence therapy response, and increase organ vulnerability while also narrowing management options. Treatment-related mechanisms include insufficient exposure (anti-drug antibodies, altered pharmacokinetics, limited tissue penetration), drug–endotype mismatch (inadequate inhibition of dominant inflammatory pathways; cytokine redundancy), and safety limitations (adverse events, absolute/relative contraindications). Disease-specific heterogeneity encompasses genetic/epigenetic susceptibility, molecular phenotypes (differences in immune cell and fibroblast populations, synovial profiles, autoantibodies), and persistent pain features. Conceptually, although the upstream drivers (e.g., comorbidities, environmental exposures, treatment-related factors) may differ across cases of D2T disease, they may converge on shared downstream signaling pathways (e.g., NF-κB, JAK/STAT, MAPK, PI3K–AKT–mTOR, HIF-1α, NLRP3), resulting in the activation of common intracellular inflammatory and stress-response programs, which might contribute to symptom persistence. Abbreviations: ACPA, anti-citrullinated protein antibody; AKT, protein kinase B; D2T, difficult-to-treat; HIF-1α, hypoxia-inducible factor 1 alpha; HLA, human leukocyte antigen; JAK, Janus kinase; MAPK, mitogen-activated protein kinase; miRNA, microRNA; mTOR, mechanistic target of rapamycin; NF-κB, nuclear factor kappa B; NLRP3, NLR family pyrin domain containing 3; PI3K, phosphoinositide 3-kinase; RA, rheumatoid arthritis; STAT, signal transducer and activator of transcription.

Although several reviews [27,33,34] offer insights into the biological basis of refractory disease, their scope has been largely limited to immunological causes of inadequate treatment response. The introduction of the overarching D2T concept provides an avenue to clarify a broad range of factors and decipher shared mechanistic processes driving management challenges in RA, including comorbidities, environmental causes, and treatment-related adverse effects. Here, we offer a more comprehensive analysis of the pathogenic drivers that might underpin the transition from standard RA to a D2T phenotype (Figure 1), examining evidence from cohorts meeting the definition of D2T RA (Table 2) as well as from illustrative studies providing insight into treatment responses and chronicity that could illuminate the determinants of D2T disease.

## 2. Methods

This narrative review aimed to synthesize current evidence on pathogenic mechanisms potentially underlying D2T RA. Relevant studies were identified through a literature search performed in multiple databases—PubMed (National Library of Medicine, Bethesda, MD, USA), Scopus (Elsevier B.V., Amsterdam, The Netherlands), and Google Scholar (Google LLC, Mountain View, CA, USA)—for English-language publications available up to December 2025. The search strategy incorporated combinations of the keywords “rheumatoid arthritis”, “difficult-to-treat”, “refractory”, “treatment resistance”, “treatment response”, and related terms.

Peer-reviewed original research articles, systematic and narrative reviews, and conference abstracts were considered, with a focus on the biological drivers of multiple targeted treatment failures and D2T disease. Additional articles were included to provide a broader clinical and biological context in the discussion of specific mechanisms. Findings were integrated using a narrative approach and organized into thematic sections.

## 3. Genetics and Epigenetics

RA has a strong genetic component, with twin studies suggesting a heritability of up to 65% [43], and genetic antecedents might also influence treatment responses, serving as a possible mechanistic basis for pathways mediating a refractory state. The human leukocyte antigen (HLA) region, located on chromosome 6, is considered the strongest genetic association for RA, although the extent of its contribution to the phenotypic variance remains controversial [44,45]. The region contains genes that encode three classes of HLA molecules playing a role in immune regulation, among which HLA class II molecules are encoded by classical (HLA-DP, -DQ, -DR) and non-classical (HLA-DM, -DO) subregions. The *HLA-DRB1* gene is recognized as particularly important in the development and progression of RA. Specific alleles that contain a motif of five amino acids at positions 70–74 of the gene, known as the ‘shared epitope’ (SE), are strongly associated with the development of anti-citrullinated protein antibody (ACPA)-positive RA [45,46]. Evidence regarding the effect of *HLA-DRB1* alleles on the effectiveness of DMARD therapy is not conclusive [47,48,49].

Multiple genome-wide association studies (GWASs) have sought to elucidate potential genetic variants associated with treatment response in RA [50,51], and a recent systematic review found 232 single-nucleotide polymorphisms (SNPs) associated with response to biological therapy [52]. However, most of these findings have not been replicated, and their clinical usefulness may be reduced by small effect size [53]. A large community-based open assessment questioned the utility of incorporating SNP information besides standard clinical characteristics with regard to the accuracy of predicting anti-tumor necrosis factor (TNF) efficacy [54]. Polygenic risk scores combining established genetic variants have been suggested as a potential tool for estimating treatment responses and determining a patient’s liability to develop D2T disease [34]. Nevertheless, a risk score incorporating 76 RA risk SNPs and 4 *HLA-DRB1* amino acid positions failed to predict response in patients who started TNF inhibitors as first-line biological therapy [49].

Epigenetic alterations are increasingly recognized as important drivers of disease progression in RA [55], and their interplay with various therapies might play a role in advancing towards a D2T state [56]. Importantly, these changes are reversible and may also be modulated by lifestyle factors such as diet and smoking [57]. For instance, smoking can influence the expression of sirtuin (SIRT) family members—histone deacetylases heavily implicated in epigenetic regulation—in RA synovial fibroblasts [58]. Specific signatures of deoxyribonucleic acid (DNA) methylation have been identified as predictors of response to conventional and biological DMARDs [59,60]. The DNA methylome signature was found to be different between early and longstanding RA [61], suggesting a potential pathway where epigenetic changes act as D2T drivers along the disease course. The role of microRNAs (miRNAs)—i.e., small non-coding ribonucleic acid (RNA) molecules that act as epigenetic regulators of gene expression—in the RA epigenome has also been extensively explored, and multiple miRNAs have emerged as possible indicators of treatment response [62].

Somatic mutations can also promote epigenetic alterations in RA [26]. A potential role for clonal hematopoiesis of indeterminate potential (CHIP) has been proposed in the context of D2T RA [26,63]. CHIP is a condition marked by the expansion hematopoietic stem cell clones carrying leukemia driver mutations without evidence of hematologic malignancy, most commonly affecting genes involved in epigenetic regulation (e.g., *DNMTA3*, *TET2*, *ASXL1*). It is closely associated with aging and promotes inflammation in non-malignant conditions. In a recent study, CHIP was associated with inferior overall survival in patients with RA, and showed associations with distinct disease phenotypes: mutated *DNMT3A* was linked to increased disease activity in seropositive RA, whereas *TET2* mutations were enriched in seronegative RA [64].

While a growing body of research has identified genetic variants and epigenetic modifications associated with disease progression and therapeutic response in RA, to date, no direct evidence has demonstrated a causal link between particular (epi)genetic factors and the development of D2T disease.

## 4. Autoantibodies

One of the hallmarks of RA is the production of autoantibodies, most importantly rheumatoid factor (RF), which recognizes the fragment crystallizable (Fc) region of immunoglobulin G (IgG), and antibodies that target post-translationally modified proteins, such as ACPAs and anti-carbamylated protein (anti-CarP) antibodies [65]. Approximately two-thirds of RA patients are seropositive. ACPA and RF are often detectable in the blood years ahead of symptoms [66], and their presence is associated with a more aggressive disease course. The development of ACPA-positive RA shows marked gene–gene and gene–environment interactions that involve smoking, SE-carrying *HLA-DRB1* alleles, and the *PTPN22* gene; in contrast, such a strong association is absent in ACPA-negative disease [67,68]. These observations support the assumption, also known as the mucosal origin hypothesis, that seropositive RA originates when genetically predisposed individuals encounter environmental exposures (e.g., smoking) that induce the production of RA-related autoantibodies in various mucosal tissues before the onset of clinically apparent disease [69].

ACPAs are thought to exert their major effector functions by forming immune complexes (ICs) and by directly binding to citrullinated proteins on cell surfaces [70]. Their pathogenic involvement may encompass the activation of osteoclasts and macrophages and the promotion of neutrophil extracellular trap (NET) formation [71]. The combined occurrence of RF and ACPAs was connected to more rapid progression to clinical disease and higher levels of pro-inflammatory cytokines [72]. While the effects of RF are poorly understood in RA, in vitro studies showed that the presence of RF enhances the capacity of ACPA-containing ICs to induce cytokine production in macrophages [73,74] and activate the complement cascade [75]. The presence of autoantibodies has been explored as a potential predictor of therapeutic response. Although autoantibody status does not appear to be associated with response to anti-TNF therapy [76,77], seropositive patients exhibited a higher effectiveness of non-TNF inhibitor biologics, including rituximab, abatacept, and tocilizumab [78,79,80]. In particular, baseline seropositivity has been consistently found to be an indicator of better response to the B-cell-depleting agent rituximab [81,82,83], attesting to the key pathogenic role of B cell activation and autoantibody production. JAK inhibitor therapy shows roughly equivalent efficacy regardless of serostatus [84,85], although data remain scarce.

Several studies have reported an increased rate of seropositivity or higher autoantibody titers in D2T RA compared with non-D2T disease [86], however, other cohorts have not replicated this association [87,88]. Overall, seropositive and seronegative RA appear to represent distinct entities, where different mechanisms may underlie unfavorable treatment outcomes, warranting stratification by serostatus in molecular research investigating D2T drivers.

## 5. Synovial Profiling and Stromal Cells

The synovium is considered the main target tissue of RA, and the emergence of new technologies over the last decade has allowed for more comprehensive molecular and histological profiling of the rheumatic joint across different disease stages and clinical activity levels. Synovial tissue studies have revealed remarkable heterogeneity among patients with RA, especially in the stromal and myeloid compartments [89]. This has prompted extensive efforts to define and characterize cell states and pathotypes in the hope of better understanding prevailing immunological mechanisms and guiding the choice of targeted treatments. Recent advances in synovium-based phenotyping of RA have been reviewed elsewhere [90,91,92].

A study by the AMP RA/SLE program identified 18 cell populations in the RA synovium that potentially act as drivers of joint inflammation [93]. The application of the corresponding cell signatures to data from patients starting anti-TNF therapy indicated that all of these signatures were higher in good responders compared with non-responders before treatment, and the signatures that decreased after treatment were mostly related to lymphocytes [94].

The increased interest in synovial pathobiology gave rise to two pioneering trials—R4RA [95] and STRAP/STRAP-EU [96]—which enabled the more direct assessment of biological agents guided by synovial features. In particular, analysis from the R4RA trial cohort revealed distinct features of a refractory subgroup characterized by the failure of ≥3 biologic classes (anti-TNF, rituximab, and tocilizumab)—i.e., corresponding to a D2T disease state—identifying a signature of >1200 unique genes and several shared pathways. This signature was linked to the fibroid/pauci-immune pathotype, which was previously associated with poor response to csDMARDs and anti-TNF [97]. Recent evidence suggests that clinically defined subgroups of refractory RA align with synovial pathotypes. In a b/tsDMARD inadequate responder cohort, patients with a persistent inflammatory phenotype—identified by high physician global assessment (PhGA) and C-reactive protein (CRP)—displayed a more lympho-myeloid and diffuse-myeloid pathotype, whereas patients with low inflammation were linked to the fibroid/pauci-immune pathotype. In a subgroup analysis of D2T-classifiable patients, an even stronger biological alignment was observed: patients in the non-inflammatory group were twice as likely to have pauci-immune synovitis compared with the inflammatory group [41].

### Synovial Fibroblasts

The stromal elements of synovial tissue, most importantly fibroblast-like synoviocytes (FLSs), present temporal and spatial heterogeneity in RA and may acquire an aggressive phenotype thought to be mainly elicited by epigenetic imprinting [89]. Considering that existing DMARDs act primarily on immune cells, stromal cell-mediated pathways could plausibly underlie treatment resistance in a proportion of patients with RA. This hypothesis is reflected by an increasing interest in the therapeutic targeting of FLSs, as reflected by preclinical studies and clinical trials [98,99]. For instance, early-phase human trials using small-molecule inhibitors of cyclin-dependent kinases (CDKs)—some of which are recognized as key regulators of FLS activity—have indicated a favorable safety profile and preliminary efficacy, paving the way for further studies [100,101].

The fibroid/pauci-immune synovial pathotype is characterized by the abundance of stromal cells and low levels of immune cell infiltration [16]. In the PEAC cohort of early RA patients, expression of fibroid gene sets correlated with less severe disease activity and radiographic progression, however, these patients showed the poorest clinical response to csDMARD therapy [16]. In a subsequent study of patients receiving treatment with certolizumab pegol, the achievement of Disease Activity Score (DAS)-28 response was significantly lower among patients with a pauci-immune histological pathotype at the baseline when compared with the lympho-myeloid and diffuse-myeloid group [102]. These findings were further broadened when assessment of the R4RA trial cohort revealed that dual non-response to tocilizumab and rituximab in patients with prior anti-TNF failure was characterized by the upregulation of fibroblast and extracellular matrix-encoding genes, including the fibroblast growth factor (*FGF*), *HOX*, and *NOTCH* family genes [97]. Nonetheless, it should be noted that after the first non-TNF inhibitor therapy within the trial, the reduction in disease activity in the fibroid pathotype was similar to other pathotypes. In a later analysis of the R4RA cohort, applying cell-type abundance phenotype (CTAP) classification to samples via bulk RNA sequencing (RNA-Seq) showed that, in line with earlier results, patients with a predicted fibroid phenotype (CTAP-F) had the lowest response to tocilizumab and rituximab, with no apparent difference between the two agents [17].

Gene expression analysis of the STRAP cohort did not show enrichment with a fibroid signature among non-responders to a first biological agent (etanercept, tocilizumab, or rituximab), although this population was less representative of D2T RA due to lower DMARD exposure [103]. However, the pauci-immune pathotype was linked to poor rituximab response in this population [96]. In a recent study of patients with ≥1 prior b/tsDMARD inadequate response, clinically non-inflammatory refractory RA (NIRRA) was linked to the fibroid/pauci-immune pathotype, and this subgroup was also characterized by less severe synovitis, fewer lymphoid aggregates, and lower immune cell markers compared with persistent inflammatory RA (PIRRA) [41].

In additional analysis from R4RA, the multidrug-resistant subgroup (with prior failure of ≥3 biologic classes) exhibited an increased baseline signature of two fibroblast subsets, namely CD34^+^ sublining fibroblasts and DKK^+^ sublining fibroblasts [97]. Furthermore, the gene encoding fibroblast activation protein (FAP) was upregulated in the deep sublining of these refractory patients [97]. Previously, the expression of FAP was found to be higher in both the synovial tissue and FLSs of active RA patients compared with those with resolved arthritis [104]. Various FAP-directed therapeutic approaches have shown promise in animal models and exploratory clinical trials [105,106], although their therapeutic value in refractory disease is yet to be determined. Another gene highly expressed across all regions of refractory patients, *CCL13* [97], encodes a chemokine known to promote the proliferation of FLSs [107].

A spatial transcriptomic analysis identified a novel subset of sublining ITGA5^+^ fibroblasts that were upregulated in active RA and in the lympho-myeloid pathotype, and their percentage showed predictive value for multiple resistance to biologics [108]. In a recent study of biopsies from patients with newly diagnosed RA who received triple csDMARD or adalimumab therapy for 6 months, pre-treatment samples of non-remission patients exhibited significant expansion of a fibrogenic fibroblast population localized to perivascular niches that was marked by high expression of cartilage oligomeric matrix protein (COMP) [109]. Interestingly, these COMP^hi^ fibroblasts served as transcriptional hubs for transforming growth factor-β (TGF-β) signaling and persisted in post-treatment samples despite successful therapeutic depletion of immune cells. The Notch signaling pathway was identified as an upstream regulator of fibroblast TGF-β activity. These results implicate the possible therapeutic targeting of TGF-β signaling in RA to attenuate synovial tissue fibrosis and prevent refractory disease [109].

## 6. Immune Cells

### 6.1. B Cells

The contribution of B cells to disease pathogenesis is multifaceted. Autoreactive B cells circulate in the peripheral blood and can be found in the synovium of patients with RA. Besides the obvious role of plasma cells in the production of autoantibodies such as RF and ACPA, B cells act as antigen-presenting cells (APCs) and secrete pro-inflammatory mediators (e.g., TNF, interleukin (IL)-6, IL-12, IL-17). Their key role of is also underscored by the clinical efficacy of rituximab, which depletes B cells by targeting the CD20 molecule located on their surface [83].

Alterations of various B cell subsets in the peripheral blood and synovium are linked to clinical outcomes with different targeted treatments. For example, higher circulating preplasma cells were associated with rituximab non-response [110]. A high baseline proportion of CD27^+^ memory B cells was linked to good clinical outcome with a first TNF inhibitor [111]. Conversely, an inverse association was found between CD27^+^ memory B cell frequency and response to rituximab in patients refractory to or contraindicated for anti-TNF therapy [112], while CCL19 level was modestly associated with treatment response in the same population [113]. In another study, the absolute number of switched memory B cells (CD27^+^IgD^−^) was significantly higher in those with worse response to rituximab, and the treatment caused a significantly stronger decline in RF serum concentration among good responders [114]. In a cohort of anti-TNF-refractory patients, a low number of double-negative (CD27^−^IgD^−^) memory B cells before rituximab treatment was found to be a predictive factor for worse response rates, and multivariate analysis further identified those with RF positivity and low double-negative B cell levels as a low-response subgroup [115]. The proportion of CD27^−^IgD^−^ memory B cells was found to be elevated in RA, and can be reduced by treatment with tocilizumab [116]. A higher percentage before therapy was associated with worse tocilizumab response [117]. A study of mostly biologic-refractory patients starting abatacept revealed that activated/memory B cells (CD38^+^IgD^+^) and unswitched memory B cells (CD27^+^IgD^+^) were expanded in good responders, and findings suggested a selective effect on memory B cells for abatacept [118].

Activated memory B cells characterized by high CD95 and low CD21 expression were found to be expanded in active RA, and exhibited relative resistance to depletion upon rituximab treatment. The CD95^+^ subset was lower at 4 months in patients with good treatment outcome [119]. Recently, the baseline proportion of the CD11c^+^CD27^−^IgD^−^ B cell population was reported to be elevated in patients who achieved clinical response to abatacept, and treatment with the drug significantly reduced its frequency [120]. Conversely, in a clinical trial of patients initiating TNF inhibitors, the frequency of CD21^−^CD27^−^IgD^−^ memory B cells was higher before treatment in the inadequate responder group, and remained elevated during follow-up [121]. Interestingly, a corresponding T-bet^+^CD21^−^CD11c^+^ population, sometimes called age-associated B cells (ABCs) or double-negative-2 B cells, shows elevated levels in the peripheral blood, synovial fluid, and synovial tissue of patients with RA [122], and correlates with disease activity [123]. It has been suggested that ABCs migrate into the RA synovium and fuel chronic inflammation by promoting FLS activation [122].

In early RA (the PEAC cohort), patients with a lympho-myeloid synovial pathotype—marked by B cell infiltration—showed the highest level of disease activity and seropositivity, and were also linked to a higher chance of biological therapy requirement at 12 months [16,124]. In patients with this pathotype, the decrease in B cell infiltration after treatment with certolizumab pegol was associated with greater improvement of disease activity [102]. The R4RA trial utilized baseline stratification for B cell status by means of synovial biopsy to compare the effect of tocilizumab and rituximab in patients with prior inadequate response to TNF inhibitors [95]. Although histological classification as B-cell–poor or B-cell–rich did not reveal major differences at 16 weeks in the response to tocilizumab vs. rituximab in either subtype, after a reclassification using RNA-Seq, tocilizumab was found to be superior to rituximab in patients with low or absent B-cell-lineage expression signature [95]. In the subsequent STRAP and STRAP-EU trials conducted in biologic-naïve patients, dichotomic stratification by synovial B cell signature did not reveal differences in response to rituximab compared with etanercept or tocilizumab in the B-cell–poor and B-cell–rich groups at 16 weeks [96].

Innovative B-cell-directed therapeutic approaches appear promising in refractory cases of RA. The utilization of bispecific T cell engagers (BiTEs) to target CD19^+^ B cells or B-cell maturation antigen (BCMA)-positive plasma cells have led to improved disease activity in case series [125,126]. Likewise, a small number of seropositive patients have shown remarkable clinical response to chimeric antigen receptor (CAR) T cell therapy directed at CD19/CD20 or BCMA antigens [127]. While these options offer a more profound depletion of the B cell compartment throughout tissues in comparison with RTX [128], more robust evidence is needed to determine their long-term safety and efficacy in RA.

### 6.2. T Cells

The critical role of T-cell-mediated immune responses is well-recognized in the development of RA but the phenotypic, developmental, and functional diversity of these cells has long posed a challenge to identifying specific disease-driving subsets. T cells, in particular CD4^+^ T cells, are the dominant lymphocytes in rheumatoid joints, yet the degree to which they contribute to chronic inflammation in established disease remains somewhat controversial [89]. While most anti-T-cell therapies have failed to demonstrate efficacy in RA, the fusion protein abatacept is an approved option recommended for patients who fail conventional drugs. Abatacept limits the CD28 co-stimulatory signal required for T cell activation by binding CD80/CD86 on APCs, although modulation of other cells (e.g., monocytes) might also account for its therapeutic benefits [129].

In a comparison of different disease states, patients with D2T RA exhibited significantly lower absolute counts of circulating total T cells, CD4^+^ T cells, and CD8^+^ T cells compared with b/tsDMARD-treated patients in remission [39]. In addition, the total T cell and CD4^+^ T cell counts were decreased in D2T RA relative to a treatment-naïve group. The measurement of CD4^+^ T cell subsets revealed that T_H1_, T_H2_, T_H17_, and regulatory T (T_reg_) cells were all lower in D2T disease than in remission, with T_reg_ cells also showing reduced levels relative to treatment-naïve RA, unlike other subsets [39]. Two-dimensional analysis of circulating T cells according to polarity and developmental stage highlighted a potential pathogenic role for effector memory T_FH_ and effector memory T_H17_ cells, whose proportion positively correlated with disease activity in DMARD-naïve RA, and decreased after treatment in patients who initiated MTX or biologics [130].

T_reg_ cells are key players in maintaining peripheral immune tolerance, and their numerical or functional decline is considered an important mechanism of losing self-tolerance in RA [131,132], with some evidence also suggesting their association with DMARD response. An increase in T_reg_ cells correlated with the reduction in disease activity in tocilizumab-treated patients [133], while an early increase in the proportion of resting T_reg_ cells after the initiation of tocilizumab was associated with lower rates of arthritis flares [134]. Good response to tocilizumab was also characterized by an increase in Helios expression in CD4^+^ T_reg_ cells upon treatment, although no such association was observed for TNF inhibitors and abatacept [135]. In patients treated with abatacept, the increase in LAG3^+^ T_reg_ cells during therapy was significantly higher in those with a major response compared with non-responders [136].

On the other hand, T_H17_ cells are increased in the peripheral blood of RA patients and chiefly express pro-inflammatory cytokines, including the IL-17 family, IL-21, and IL-22 [137]. Inadequate response to anti-TNF therapy has been linked to a higher circulating T_H17_ cell frequency at baseline, and non-responders tend to exhibit an expansion of T_H17_ cells after treatment [138,139], although one study found a correlation between increasing levels and ultrasonographic improvement [140]. Gene expression analysis of T_H17_-enriched CCR6^+^ T cells from patients initiating infliximab identified the transcription factor USF2 as an upstream regulator of increased pro-inflammatory signaling pathways among non-responders. Silencing of *USF2* led to decreased expression of cytokines including IL-17A, IL-22, interferon (IFN)-γ, TNF, and granulocyte-macrophage colony-stimulating factor (GM-CSF) in pathogenic T_H17_ cells [141]. T_H17_ cells are the main source of IL-17A and IL-17F, which act synergistically with other cytokines (e.g., TNF, IL-1β) to mediate chronic inflammation in target tissues [142,143]. In clinical trials, IL-17-directed approaches have produced only modest improvements in RA outcomes and have failed to outperform established targeted treatments. In contrast, a proof-of-concept study found that add-on therapy with a dual IL-17A/17F inhibitor in patients with inadequate response to certolizumab pegol led to a rapid decrease in disease activity [144], suggesting that the IL-17 pathway might be upregulated in some refractory cases and is not entirely redundant for therapeutic targeting.

The pathogenic role of imbalance between T_H17_ and T_reg_ cells is further supported by the finding that T_H17_/T_reg_ ratio was higher in both D2T RA and non-D2T RA patients when compared with a treatment-naïve group or healthy controls [39]. The majority of currently available biologics seem to actively interfere with T_H17_ and T_reg_ cells [137], supporting the assumption that restoring their balance might be beneficial in RA management. The therapeutic administration of low-dose IL-2 appears clinically effective and safe in RA, while also leading to an increase in the levels of T_reg_ cells and a decreased T_H17_/T_reg_ ratio [145]. Notably, the effects of low-dose IL-2 on lymphocyte subsets have also been observed in a patient group meeting the D2T RA criteria [39].

Follicular helper T (T_FH_) cells, a subset often identified as CXCR5^+^PD-1^+^CD4^+^, usually reside in secondary lymphoid tissues but are rarely observed in the synovium. In RA, they induce the differentiation of autoantibody-producing B cells by directing the formation of germinal centers [146]. A smaller population of similar cells, called circulating T_FH_ cells, can also be found in the blood, and may exhibit altered levels or phenotypes in autoimmune disease [147]. Abatacept therapy leads to a marked reduction in circulating T_FH_ cell proportions in RA, and a higher baseline proportion has consistently been linked to favorable clinical responses to abatacept [116,148,149]. On the other hand, a higher T_FH_ frequency at baseline predicted poor response to anti-TNF treatment in a seropositive RA cohort [149], although earlier studies in RA patients reported no significant association [116,148].

A comprehensive analysis further established a CXCR5^−^PD-1^+^CD4^+^ population that was markedly expanded in the synovial tissue and fluid of seropositive RA patients and could also be detected in peripheral blood. These cells, subsequently coined peripheral helper T (T_PH_) cells, supported B cell activation in vitro in an IL-21- and SLAMF5-dependent manner, similar to T_FH_ cells [150]. A recent study suggested that they exhibit two distinct functional stages in RA: stem-like T_PH_ cells reside in tertiary lymphoid structures and promote the autoantibody production of B cells, while effector-like T_PH_ cells migrate out of these structures and express effector molecules [151]. Fine cellular analysis of RA synovial tissue revealed a distinct association between the proportions of T_PH_ cells and CD11c^+^LAMP1^+^ ABCs [17]. A study of synovial tissue T cell clusters suggested a phenotypic progression of T_PH_ cells during the disease trajectory: treatment-naïve samples showed predominance of a phenotype expressing *CCL5*, whereas classical CXCL13^+^ T_PH_ cells dominated in later stages, particularly in D2T RA [40]. In a study of seropositive patients initiating abatacept or TNF inhibitors, circulating T_PH_ cells significantly decreased in both groups after treatment, although their baseline frequency was not associated with clinical response to either drug [149].

CX3CR1^+^CD4^+^ T cells have recently been identified as a cytotoxic T cell subset associated with elderly-onset RA, and they showed increased proportions in the peripheral blood of patients with D2T RA compared with non-D2T disease [35]. Moreover, the expression levels of programmed cell death protein 1 (PD-1) and CD38 by these cells were significantly elevated in the D2T group, suggesting their potential role as markers of a treatment-resistant T cell population [35].

### 6.3. Myeloid Cells

Macrophages promote inflammation and activate other cell types, such as FLSs and T cells, by producing a wide range of cytokines, and the degree of synovial macrophage infiltration correlates with the severity of joint erosion [152]. These cells exhibit elevated numbers in active disease, and their depletion by different DMARDs has been linked to therapeutic success. An early study reported an association between good response across various csDMARDs and a post-treatment reduction in sublining macrophages [153]. A significant fall in the amount of sublining macrophages between baseline and 12 weeks was observed among good responders to certolizumab pegol, and the post-treatment change in these cells correlated with the change in DAS28 score [102]. The numbers of sublining macrophages, along with tissue-resident and tissue-infiltrating subsets, were reported to be higher before treatment in infliximab responders [154]. A synovial gene expression signature corresponding to the myeloid phenotype (dominated by inflammatory macrophages) was associated with better response to anti-TNF therapy in established RA [155]. Moreover, high serum soluble intercellular adhesion molecule 1 (sICAM1) level was linked to the myeloid phenotype, and patients with high sICAM1/CXCL13 ratio showed a higher response to TNF inhibitors than those with a lower ratio, whereas an inverse relationship was observed with tocilizumab [155]. A low sICAM1/CXCL13 ratio was also reported in filgotinib responders after bDMARD failure [156].

The change in synovial macrophages correlated with DAS28 response after rituximab treatment [157]. Evidence from the R4RA trial points to synovial myeloid cell infiltration as a driver of response to tocilizumab but not rituximab: responders to tocilizumab showed significantly higher levels of macrophage–monocytes and myeloid dendritic cells (mDCs) than non-responders, and mDC-rich individuals exhibited a higher response rate to tocilizumab compared with rituximab. Tocilizumab was also more effective than rituximab in patients with a combination of low B cells and high macrophages/mDCs [97]. Furthermore, non-responders failed to reduce CD68^+^ sublining macrophages after tocilizumab therapy, as opposed to responder patients [97].

Single-cell RNA-Seq profiling of synovial tissue macrophages revealed that a MerTK^+^CD206^+^ subset was increased in healthy donors as well as in sustained remission (under MTX and TNF inhibitors) when compared with treatment-naïve and MTX-resistant active RA [158]. Furthermore, a low proportion of MerTK^+^CD206^+^ macrophages and a low MerTK^+^CD206^+^/MerTK^−^CD206^−^ ratio predicted disease flare among those who tapered and discontinued biological treatment after remission. Additional analysis suggested that MerTK^+^ cells produce resolvins, especially in a remission state, and can induce reparative responses in FLSs. On the other hand, MerTK^−^ cells in active RA are marked by the expression of alarmins and osteopontin, which produce pro-inflammatory cytokines (e.g., TNF, IL-6), leading to increased FLS expression of chemokines and matrix metalloproteinases (MMPs) [158], indicating a key role of MerTK-associated macrophage–FLS crosstalk in the regulation of synovial immune homeostasis [159]. In a gene expression analysis of the R4RA cohort of anti-TNF inadequate responders, *MERTK* was upregulated in lympho-myeloid and diffuse-myeloid patients, and positively correlated with immune cell infiltrates, whereas no such differences were observed in early RA patients (PEAC cohort) [160], overall suggesting that DMARD exposure and/or prolonged inflammation might influence MerTK expression. The SPP1^+^ cluster of MerTK^−^ macrophages expresses osteopontin, which has bone-resorbing properties [158]. In a synovial fluid analysis, the pre-treatment level of osteopontin was higher in the synovial fluid of early American College of Rheumatology 20% (ACR20) responders to TNF/JAK inhibitors, and SPP1^+^ macrophage proportions and osteopontin levels correlated with DAS28 [161]. In the same study, more prolific communications between macrophages and T cells predicted a better response to therapy [161].

Dendritic cells (DCs) are implicated in RA pathogenesis, and different phenotypes may substantially influence immune tolerance. An analysis of synovial tissue myeloid DC subsets across disease stages found that AXL^+^ type 2 DCs (DC2s) dominated in the healthy synovium, constituting about 40% of all DCs. This population was marked by the expression of the immune checkpoint *AXL* and other genes involved in immune tolerance and tissue repair. The AXL^+^ DC2 cluster showed a significant reduction in active RA, with the lowest proportions found in D2T samples, and exhibited no recovery in sustained remission [40].

Dendritic cell precursors (pre-DCs), a relatively newly identified cell subset that differentiates into type 1 and type 2 conventional DCs, appear to be capable of naïve CD4^+^ T cell stimulation and the secretion of cytokines such as IL-12 and TNF [162]. Gene expression analysis of the peripheral blood immune cells of patients with active RA revealed that an increase in pre-DCs before the initiation of a new DMARD predicted treatment resistance at 6 months, and this pre-DC gene signature was a better predictor of response than ACPA status or disease duration [163]. Furthermore, type I IFN signaling inversely correlated with pre-DC gene expression [163].

### 6.4. Neutrophils

Neutrophils are the most abundant cell type detected in the synovial fluid of RA patients, and can also be found in rheumatoid synovial tissue [164]. They are believed to exert their pathogenic effects through the release of inflammatory mediators, the formation of NETs, and the modulation of innate and adaptive immune responses [164]. RA patients exhibit a higher neutrophil-to-lymphocyte ratio (NLR) in their peripheral blood [165], and a higher ratio at baseline was associated with better responses to IL-6 inhibitors and JAK inhibitors [166,167,168]. However, studies show conflicting results on the association of NLR and TNF inhibitor treatment outcomes [167,169,170]. In an RNA-Seq analysis of blood neutrophils from RA patients before starting TNF inhibitors, a total of 23 neutrophil transcripts demonstrated predictive value for treatment response, of which 10 IFN-regulated genes predicted good response, while 13 genes encoding neutrophil granule proteins predicted non-response [171]. A study on a large cohort of RA patients with prior anti-TNF therapy identified circulating neutrophil activation markers as predictors of subsequent baricitinib response, with a combination of low levels of calprotectin and neutrophil elastase–DNA complexes corresponding to worse ACR20 response [172].

Direct evidence linking synovial neutrophils to refractory disease is limited. RNA-Seq of paired blood and synovial fluid neutrophils from patients with high disease activity revealed an altered pro-inflammatory phenotype in the synovial fluid that was marked by delayed apoptosis, the production of reactive oxygen species (ROS), and NET release [173], which may drive chronic inflammation and structural damage. The presence of neutrophilic infiltration in synovial tissue was linked to higher disease activity [174].

## 7. Pain and the Nervous System

### 7.1. Pain Pathways

**Figure 2:**
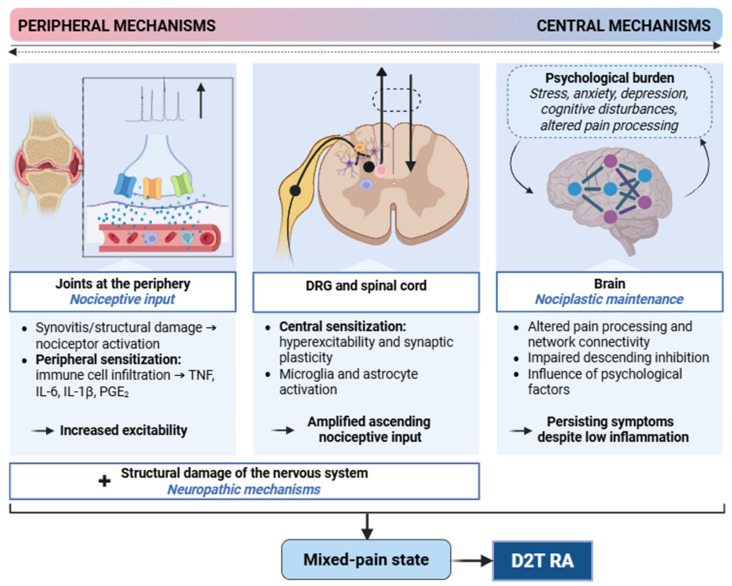
The interaction of peripheral and central mechanisms contributing to a mixed-pain state in difficult-to-treat (D2T) rheumatoid arthritis (RA). In the RA joint (**left**), synovitis and/or structural damage activate nociceptors, while immune cell infiltration and inflammatory mediators (e.g., TNF, IL-6, IL-1β, PGE_2_) promote peripheral sensitization and increased excitability. In the dorsal root ganglia and spinal cord (**middle**), sustained afferent input triggers central sensitization characterized by dorsal horn hyperexcitability and synaptic plasticity, supported by microglial and astrocyte activation, thereby amplifying ascending nociceptive signals. In the brain (**right**), altered pain processing and network connectivity, together with impaired descending inhibition, contribute to nociplastic maintenance of pain. Mental health alterations (i.e., stress, anxiety, depression, cognitive disturbances) can further influence central pain processing. In addition, structural damage of the somatosensory nervous system may introduce neuropathic mechanisms. The coexistence of nociceptive, nociplastic, and (when present) neuropathic components results in a mixed-pain state, which can sustain patient-reported symptoms even when objective inflammation is low, thereby contributing to persistent symptom burden and the D2T clinical phenotype. Abbreviations: DRG, dorsal root ganglia; PGE_2_, prostaglandin E_2_; TNF, tumor necrosis factor; IL, interleukin.

Pain is a cardinal symptom of RA that may occur despite adequate therapeutic control of disease activity, thereby reducing quality of life and promoting a D2T state. Mechanistically, three main types of pain can be distinguished: *nociceptive* pain reflects nociceptor activation by damage to non-neural tissue; *neuropathic* pain results from a lesion or disease of the somatosensory nervous system; and *nociplastic* pain arises from altered nociception without clear evidence of tissue damage driving peripheral nociceptor activation or of a somatosensory system lesion [175]. Persistent pain in RA cannot be explained by a single mechanism, instead, it represents a mixed-pain state arising from different inflammatory and nociceptive mechanisms at the periphery linked to maladaptive central pain-processing alterations that, overall, lead to a pain level that is often disproportionate to synovitis (Figure 2).

#### 7.1.1. Peripheral Drivers

Joint pain signals usually begin with the activation of specialized peripheral sensory neurons arising from the dorsal root ganglia, whose terminals innervate the synovium, periosteum, ligaments, and adjacent connective tissues and, in the event of noxious damage, transmit stimuli to the central nervous system (CNS). In inflammatory states, these afferents may become spontaneously active and/or exhibit a greater response to mechanical or chemical stimuli, overall referred to as peripheral sensitization. An important mechanism behind this process is that mediators released by inflammatory cells (e.g., prostaglandins, TNF, IL-6, IL-1β) infiltrating the joint can directly act on nociceptors, thereby modulating their activation and firing frequency [175]. Besides, the recruitment of ‘silent’ (mechanically insensitive) nociceptors, which are quiescent under baseline conditions but become mechanosensitive in inflammation, may contribute to persistent tenderness and lowered pressure pain thresholds [175,176]. Importantly, these neuron-level changes, together with ongoing pain, may persist after the reduction of visible swelling and may be maintained by different mediators within the joint niche [175].

Synovial fibroblasts are increasingly recognized as peripheral contributors of pain. Fibroblast-derived mediators have been linked to amplifying sensory neuron activation [177], and a peptidase inhibitor 16-expressing fibroblast population has been implicated as a contributor to pain-like phenotypes in animal models [178,179]. A recent study proposed that fibroblasts enhance sensory nerve sprouting in the RA joint. The work identified a module of pain-associated genes in patients with low synovial inflammation, most of which were most robustly expressed by synovial lining fibroblasts. Further analysis suggested that fibroblast genes enhance the growth of calcitonin gene-related peptide (CGRP)-positive pain-sensing neurons into synovial hypertrophic papilla, with netrin-4 emerging as a potential mediator [180]. Interestingly, patient-reported pain levels were similar in low vs. high synovial inflammation, and pain scores were associated with cell density in high-inflammatory but not low-inflammatory synovium [180]. In a cohort of b/tsDMARD inadequate responders, patients with PIRRA—defined as high PhGA and CRP—reported pain levels comparable to those in NIRRA, despite the latter group predominantly exhibiting a fibroid/pauci-immune pathotype [41].

The observation that pain-like behavior can be induced by RA-related autoantibodies in experimental settings, even in the absence of overt inflammation, also supports the concept of peripheral neuronal modulation by immune effectors beyond synovitis-driven nociception [181,182]. ACPAs have been reported to induce mechanical hypersensitivity under conditions of limited overt synovitis, and antibody-mediated effects on osteoclast activity and Fc receptor signaling have been implicated [182,183]. In addition, cartilage-binding antibodies—e.g., against collagen type II (CII) or COMP—can trigger pain-like behavior that shows only partial concordance with inflammatory markers [184]. While further validation and translation to clinical stratification remain challenging [175], this line of work strengthens the rationale that several peripheral factors, beyond local joint inflammation, may contribute to persistent pain phenotypes, consistent with cases of D2T RA marked by ongoing symptoms despite a reduction in inflammatory activity.

#### 7.1.2. Central Drivers

In addition to peripheral processes, CNS alterations are also involved in pain modulation. Central sensitization can be described as the increased responsiveness of nociceptive neurons in the CNS, which might result in hyperalgesia (i.e., an elevated level of pain in response to noxious stimuli) and/or allodynia (i.e., the experience of pain in response to normally non-painful stimuli) [185]. These processes develop after activation by peripheral noxious stimuli, when primary afferent nociceptors release glutamate from their central terminals in the spinal dorsal horn, which binds to N-methyl-D-aspartate (NMDA) receptors on postsynaptic neurons. In parallel, other mediators—including substance P, CGRP, and brain-derived neurotrophic factor (BDNF)—are also released, further influencing synaptic transmission. Other cell types, such as microglia and astrocytes located in the spinal cord and brain, also play a key role in central sensitization, together with infiltrating macrophages and lymphocytes, potentially further amplifying these processes. After becoming activated, they produce additional mediators (BDNF, lipid mediators, etc.), cytokines (TNF, IL-1, etc.), and chemokines (CCL2, CXCL1, etc.), further influencing synaptic plasticity and exerting pain-modulating effects [185,186]. A recent transcriptomic analysis of peripheral blood mononuclear cells (PBMCs) comparing D2T and non-D2T patients revealed alterations linked to neuroinflammatory pathways, further supporting the role of CNS processes in the D2T state. For example, the gene encoding neuregulin-1 (a member of the epidermal growth factor family) was upregulated in the D2T group; in turn, downregulated genes included *NEGR1* (neuronal growth regulator 1) as well as *S100B* (which encodes a calcium-binding protein chiefly expressed in the CNS) [36,37]. Interestingly, serum S100B levels negatively correlate with cognitive function scores in RA [187].

Neuroimaging evidence further underscores CNS involvement in disease pathogenesis and related chronic pain. Functional magnetic resonance imaging (fMRI) studies in RA revealed altered activation and connectivity patterns across several brain regions, mainly affecting the thalamus, insula, anterior cingulate cortex, and medial prefrontal cortex (mPFC), as well as large-scale network-level alterations, chiefly in the default mode (DMN), salience, and frontoparietal networks [188,189,190,191]. A study using resting-state fMRI revealed changes in the connectivity and activity patterns of the somatosensory area and the posterior cingulate cortex, an important node of the DMN, among D2T RA patients. Furthermore, after acute pain stimulation, the D2T group exhibited differential alterations in connectivity patterns compared with non-D2T patients and healthy individuals [37]. TNF blockade has been shown to rapidly normalize pain-related brain activity (often within 24 h), preceding improvement in joint swelling, which suggests a ‘bottom-up’ contribution of peripheral cytokine signaling to central sensitization [192]. In contrast, the fact that symptoms persist in many patients even after effective immunosuppression likely reflects ‘top-down’ processes, such as impaired descending inhibition and network rewiring, consistent with nociplastic pain mechanisms [193]. The dysregulation of conditioned pain modulation, reflecting inefficient descending inhibition, was associated with poor EULAR response to subsequent DMARD therapy [194].

Together, the convergence of peripheral immune mechanistic drivers and altered CNS processes may be accountable for pain persistence and highlights the importance of recognizing the CNS as a complementary therapeutic target in RA, especially in D2T disease [193].

### 7.2. Fatigue

Fatigue is a highly prevalent and disabling symptom in RA, affecting up to 80% of patients. A well-known framework of fatigue in RA encompasses disease-related factors (e.g., inflammation, pain, disability), personal factors (e.g., work, health issues), and cognitive-behavioral concepts (e.g., thoughts, feelings, behaviors) [195]. In two cohorts, patients with D2T RA reported significantly higher fatigue scores, along with more pain and higher disability, in comparison with the non-D2T groups [196,197]. In a sex-stratified analysis, female patients with D2T RA showed higher fatigue levels, while men exhibited no such differences [197]. Altered brain connectivity patterns that were associated with high inflammatory markers—involving the inferior parietal lobule, mPFC, and large-scale networks such as the DMN and the dorsal attention network—predicted not only pain but also fatigue and cognitive dysfunction in patients with RA [191], suggesting shared neurobiological pathways for these symptoms.

### 7.3. Fibromyalgia and Mental Health

FM is primarily characterized by widespread pain along with other somatic symptoms, corresponding to a prototypical nociplastic pain state marked by altered nociception in the absence of clear ongoing tissue damage or somatosensory system lesion. Comorbid FM, estimated to affect up to 30% of RA patients [198], can substantially amplify not only pain perception but also fatigue and disability, thereby leading to the overestimation of disease activity [199]. Patients with RA who had high clinical ‘fibromyalgianess’ scores exhibited altered connectivity between the DMN and the insula (akin to previous findings in FM), even without meeting the FM diagnostic criteria [188], supporting an overlap in pathogenesis. Importantly, FM was reported to be independently associated with the D2T RA phenotype [200].

Depression and anxiety often occur in parallel with RA and are usually associated with more severe symptoms, including higher pain intensity and poorer response to treatment [201], with some data showing that they are more frequently present among D2T patients [87,196]. In addition to mood disorders, psychological stress also represents a substantial burden in RA, and higher stress levels have been linked to greater pain and disability and lower social support [202]. Pain catastrophizing is consistently associated with higher pain, worse function, and poorer quality of life, and can also inflate disease activity by influencing patient-reported outcomes [203]. These mechanisms are directly relevant to D2T RA, where EULAR points to consider emphasize the holistic assessment of persistent symptoms and comorbidities to avoid misclassifying pain-dominant trajectories as inflammatory failure [23].

### 7.4. The Inflammatory Reflex

A growing body of research suggests that a diverse family of reflex neural circuits contributes to immunological homeostasis [204]. These include the so-called inflammatory reflex, which is initiated when vagus nerve afferents detect peripheral inflammation (e.g., TNF, IL-6, IL-1β), leading to reflexive activation of the cholinergic anti-inflammatory pathway, whereby signals are transmitted from vagal efferents via the splenic nerve to a specialized choline acetyltransferase-positive T cell population in the spleen. These T cells release acetylcholine, which binds the α7 nicotinic acetylcholine receptor (α7nAChR) on splenic macrophages and other immune cells, resulting in broad downregulation of intracellular signaling pathways (NF-κB, JAK/STAT, inflammasome) and a subsequent decrease in the production of pro-inflammatory cytokines [205,206].

Established RA is characterized by autonomic imbalance, as supported by findings of diminished heart rate variability and altered catecholamine levels [207]. A study reported decreased parasympathetic activity in individuals at risk for RA, and α7nAChR expression levels were lower in those with a high resting heart rate, overall suggesting that inflammatory reflex dysregulation precedes the onset of disease [208]. Rodent models of RA have demonstrated that electrical stimulation of the vagus nerve can modulate systemic inflammation [209], leading to the assumption that targeted vagus nerve stimulation (VNS) might offer therapeutic benefits in RA. Following encouraging results from early clinical studies using active stimulation, larger trials were launched to determine the efficacy of VNS. In a cohort of b/tsDMARD-naïve patients, auricular stimulation failed to meaningfully improve disease activity compared with sham stimulation [210]. However, a recent large sham-controlled trial evaluating an implantable VNS device met its primary efficacy endpoint and demonstrated durable clinical improvement in patients with prior exposure to ≥1 targeted treatment, 43% of whom were classified as having D2T RA [206].

## 8. Smoking

Tobacco exposure shows a pronounced effect on RA development, and smoking accounts for about 20–30% of the environmental risk for RA [211]. As above-mentioned, smoking is more strongly associated with ACPA-positive disease, especially in carriers of the shared epitope [211]. It can exacerbate systemic immune responses in RA [212], as evidenced by higher serum levels of pro-inflammatory cytokines (e.g., TNF, IL-2, IL-6, IL-12, IFN-γ) and increased disease activity among current smokers [213]. Furthermore, it has been demonstrated that smoking can directly influence gene expression in the RA joint, potentially activating pathways that promote chronic inflammation [214].

Smoking is a predictor of MTX inadequate response [215], and multiple studies indicate that it might also be associated with poor response to biologics [216], especially TNF inhibitors [217,218], although findings are inconsistent [219,220]. In an early RA cohort, baseline current smokers needed more DMARD combinations or biologics within three years [221]. Smokers showed higher levels of TNF and an increased ratio of TNF/soluble TNF receptor (sTNFR) released by stimulated T cells in a study of patients with established RA, and these also showed a relationship with the extent of smoking [222], possibly contributing to reduced anti-TNF responses. The production of anti-drug antibodies (ADAs) has been proposed as a mechanism underlying worse bDMARD response among smokers. In a cross-sectional study of inflammatory arthritis patients receiving TNF inhibitors, those with neutralizing ADAs were more frequently smokers and exhibited worse disease outcomes [223]. Similarly, lifetime smoking carried a higher risk of ADA development among infliximab-treated RA patients [224]. A multicohort study of patients with autoimmune conditions—including RA—found that tobacco smoking was positively associated with time to develop ADAs after treatment with different biopharmaceuticals [225].

While most observational studies found no substantial difference between D2T and non-D2T RA groups in terms of smoking history [86], smoking was associated with a poly-refractory group of D2T RA who failed all b/tsDMARD classes [32]. Patients with refractory RA showing objective evidence of joint synovitis had higher smoking history than patients with no inflammation [226]. Furthermore, smoking was more frequent in a subset of D2T RA patients with pain syndromes and obesity [196], and multiple studies suggest a link between smoking habits and the appearance of various extra-articular manifestations in RA [216]. These findings underscore the complex interplay of smoking with other D2T contributors.

## 9. Nutrition and the Digestive System

The mucosal origin hypothesis postulates that RA is initiated at mucosal sites, such as the mouth, gut, and lungs, as a result of altered crosstalk between the mucosal immune system and local microbiota [227]. Increasing evidence implicates microbial dysbiosis—especially in the oral cavity and intestine—in abnormal immune responses [228], which may continue to influence disease course in established RA, thereby contributing to suboptimal treatment outcomes and D2T RA. Several risk factors, including diet, smoking, and obesity, might exert some of their effects on disease progression by altering the digestive microbiome [229].

### 9.1. Periodontal Health

Numerous studies indicate a close relationship between periodontal disease and RA, corroborated by the model that *Porphyromonas gingivalis*, a periodontal pathogen, can induce protein citrullination at inflamed local sites by expression of the enzyme peptidylarginine deiminase (PPAD), resulting in the production of ACPAs [230]. RA and periodontitis are characterized by an overlapping molecular landscape, including an upregulation of pro-inflammatory cytokines (e.g., TNF, IL-6, IL-1) at the level of target tissues [231]. Patients with low baseline anti-PPAD titers exhibited a better clinical response to bDMARD treatment, which might be explained by a lower degree of protein citrullination [232]. A higher level of periodontal inflammation was linked to reduced DAS28 response after biological therapy [233].

Non-surgical periodontal treatment appears to have a beneficial effect on RA outcomes, although some uncertainty remains [234]. In a small study of seropositive patients with resistance to multiple biologics, periodontal therapy led to DAS28 response in five out of eight participants after 3 months [235], suggesting a possible adjunctive strategy for D2T disease. In turn, despite earlier assumptions, accumulating evidence indicates that most DMARDs exert positive effects on periodontitis, further supporting the presence of shared pathogenic processes in the two conditions [234]. The interplay between treatment responses and periodontal status in established RA warrants further investigation into mechanistic links.

### 9.2. Gut Microbiota

RA patients exhibit a dysbiotic phenotype of the intestinal microbiome, and composition of the gut microbiota differs across disease stages and clinical activity states [228], although it has been challenging to elucidate causal relationships between RA pathogenesis and altered microbial profiles [236]. Emerging evidence suggests that DMARDs are capable of restoring the microbiome to a more eubiotic state, especially in those who clinically respond to therapy, and several recent works have proposed models utilizing gut microbiome signatures to predict subsequent response to various therapies [237,238]. In addition, RA has been linked to impaired intestinal barrier function, and the change in disease activity after DMARD initiation correlated with a reduction in serum gut permeability markers. Furthermore, biologic responders displayed a significant decrease in these markers, unlike non-responders and csDMARD-treated patients [239]. Supplementation with various probiotics has shown benefits in reducing inflammation in RA, although more evidence is needed on their efficacy [240].

In a recent study comparing the gut microbiota of D2T RA patients and those with stable disease under a single csDMARD, the two groups exhibited similar α- and β-diversity metrics but differed in abundance of some features. The phylum Proteobacteria, as well as the families Lachnospiraceae and Pasteurellaceae—and their respective genera *Coprococcus* and *Haemophilus*—were less abundant in D2T RA [42]. Remarkably, Lachnospiraceae are major producers of short-chain fatty acids (SCFAs), which are thought to exert anti-inflammatory effects [241], and a high fecal abundance of SCFA-producing genera was observed in patients treated with TNF inhibitors [242]. On the other hand, the phylum Bacteroidetes and the genus *Megasphaera* were found to be enriched in the D2T group, and multivariate analysis revealed that a higher Firmicutes/Bacteroidetes ratio was associated with a reduced risk of D2T RA [42]. This ratio was previously found to be reduced in RA [243], although another work reported a higher pre-treatment ratio among MTX non-responders [244].

### 9.3. Dietary Factors

The potential influence of nutrition and dietary habits on RA progression have received increased attention in recent years [245]. For instance, high dietary sodium intake has been linked to increased RA risk, especially among smokers [246], and studies utilizing animal models and human cells have suggested that high-salt conditions induce the expression of serum/glucocorticoid-regulated kinase 1 (SGK1), leading to increased production of pathogenic T_H17_ cells [247,248]. High sodium intake was also associated with an increased prevalence of major adverse cardiovascular events (MACE) among RA patients [249], although a recent study suggested that, up to a certain threshold, a rise in sodium intake confers protection against all-cause mortality in RA [250].

The role of nutrition in the D2T phenotype remains unknown; one study found similarly high adherence rates to the Mediterranean diet in both D2T and non-D2T groups [42]. To date, no clinical trials have evaluated the efficacy of specific dietary therapeutic approaches in the D2T population [7]; nonetheless, various diet interventions might lead to improved treatment outcomes in RA [251]. For instance, a randomized controlled trial reported that fish oil supplementation decreased the likelihood of triple csDMARD failure in early disease [252].

## 10. Comorbidities

Concomitant diseases are highly prevalent in RA [253] and represent key contributors to D2T disease, with most cohort studies reporting a higher comorbidity burden among patients with D2T RA [86]. Some conditions can directly influence RA pathogenesis by perpetuating a pro-inflammatory state, while others complicate disease management chiefly by limiting the choice of therapy or confounding disease assessment [254,255]. Effective management of comorbidities requires close collaboration among specialties; nevertheless, polypharmacy might also contribute to adverse RA outcomes, as suggested by a finding that each additional prescription for a comorbid condition in RA reduces the clinical response to biologics by 8% [256]. The rheumatic disease comorbidity index (RDCI) was identified as an independent risk factor for mortality in a D2T RA cohort [257].

### 10.1. Obesity and Diabetes

Obesity is considered a risk factor for RA onset [258], and it has also been reported to be independently associated with the development of D2T RA [200]. Adipose tissue can function as an active endocrine organ, and excessive fat accumulation promotes a chronic low-grade inflammatory state [258], whereby increased numbers of adipocytes and infiltrating immune cells influence the release of bioactive substances, including classical pro-inflammatory cytokines (e.g., TNF, IL-6) and adipokines (e.g., leptin, resistin, adiponectin), which might further amplify systemic immune activation [259]. Adipokine levels tend to be elevated in RA, and some targeted therapies, particularly IL-6 receptor inhibitors, might be capable of modulating adipokine profiles, although the findings are inconsistent [260,261]. Meta-analyses indicate that a high body mass index (BMI) negatively affects the outcome of TNF inhibitor treatment [262,263], and non-responders were found to display higher serum leptin levels [264]. While findings on other bDMARD classes are more conflicting [262,265], a study of patients with prior anti-TNF failure reported worse response rates to a second-line non-anti-TNF biologic among obese individuals [266]. Emerging data suggest that JAK inhibitors remain effective regardless of BMI status [261,267], implying a possible advantage over biological agents in patients with high BMI.

The influence of obesity might extend beyond inflammation. Paradoxically, multiple imaging studies have indicated that a higher BMI is associated with lower radiographic joint damage and less severe synovitis, despite higher scores of clinical disease activity [268,269]. This might be explained by decreased adiponectin levels in obese patients [268]; moreover, obesity appears to interfere with the clinical grading of disease activity [270]. A synovial tissue analysis found that treatment-naïve patients with overweight/obesity displayed higher rates of follicular synovitis and higher levels of resident sublining immune cells (CD68^+^, CD21^+^, and CD20^+^) than those with normal weight; however, no such differences were detected among MTX inadequate responders, while overweight/obese patients in stable remission under MTX and TNF inhibitor therapy showed a higher degree of residual synovitis [271]. Altered pharmacokinetics, such as decreased absorption, might also contribute to poor treatment outcomes in obese patients [272].

RA is associated with an increased risk of diabetes mellitus [273], and some cytokines, such as TNF and IL-1β, serve as overlapping drivers of inflammation in both diseases [258]. According to a recent study, patients with D2T RA are more likely to have diabetes [274]. Monocytes from patients affected by both RA and type 2 diabetes (T2D) exhibited an increased production of IL-1β via NLRP3 inflammasome activation compared with individuals who only had one of the conditions [275]. In an open-label clinical trial of patients with concomitant RA and T2D, the IL-1 inhibitor anakinra effectively reduced both hemoglobin A1c (HbA1c) and RA activity, as opposed to anti-TNF therapy, which failed to achieve glycemic control [276]. Oral GC therapy is an important risk factor for diabetes, and the risk of incident diabetes might be influenced by higher dosage and longer treatment duration in RA patients [277], which is particularly relevant for patients unable to taper GCs.

### 10.2. Cardiovascular Disease

Patients with RA have a significantly elevated risk for developing CVD compared with the general population, and recent findings indicate that comorbid CVD is more frequent in D2T RA [87,278]. CVD represents the leading cause of death in RA, being responsible for over 50% of premature mortality [254]. The complex interplay between RA and CVD is thought to be mediated by overlapping pathogenic mechanisms that, in many cases, are further modulated by traditional CVD risk factors (e.g., obesity, smoking, hypertension, dyslipidemia, and diabetes mellitus) [279]. Research on the prevailing mechanisms driving CVD risk and progression in the D2T RA state is still limited.

The inflammatory state in RA, marked by the sustained elevation of cytokines such as TNF, IL-1, and IL-6, promotes the progression of atherosclerosis and plaque vulnerability [280]. Patients with RA exhibit endothelial dysfunction in both the macrovascular and microvascular beds, leading to higher oxidative stress and the upregulation of adhesion molecules [281]. Endothelial progenitor cells (EPCs) and angiogenic T cells (T_ang_), involved in vascular repair mechanisms, were found to be reduced in RA, with T_ang_ levels even lower in patients with comorbid CVD [282]. Systemic inflammation in RA has also been linked to lipid alterations, including impaired cholesterol efflux by high-density lipoprotein (HDL) particles [283], as well as increased macrophage uptake of oxidized low-density lipoprotein (LDL) resulting in foam cell formation [284]. Notably, multiple studies indicate the existence of a ‘lipid paradox’ in active RA, whereby lower levels of total cholesterol and LDL are associated with an elevated CVD risk [285]. Positivity for ACPA and RF has been linked to an increased risk of CVD, even in the absence of RA [279]. In a cohort study assessing cardiovascular events among RA patients, an elevated T_H17_/T_reg_ ratio was associated with a higher rate of coronary artery disease (CAD), while absolute counts of the two subsets did not display such an association. The link between T_H17_/T_reg_ ratio and CAD risk was even more pronounced in a subgroup of patients refractory to ≥2 b/tsDMARDs, and this was primarily mediated by T_reg_ cells, whose absolute counts were also associated with CAD rates [286].

The adequate control of disease activity with anti-rheumatic drugs is expected to reduce the occurrence of cardiovascular events, as reinforced by the EULAR recommendations for risk management [287]. However, some RA medications can act as double-edged swords by activating mechanisms that lead to adverse cardiovascular outcomes, which might complicate RA management, particularly in the presence of additional CVD risk factors [288]. For instance, GCs may affect cardiovascular health by impairing endothelial function and altering lipid metabolism, and some observational studies reported increased rates of cardiovascular events among GC users, particularly with higher doses. However, evidence on the association of GC treatment and CVD risk remains conflicting [289]. Switching to a JAK inhibitor after the failure of multiple biologics appears beneficial in the D2T population [290], however, post hoc analyses from a tofacitinib clinical trial have raised safety concerns regarding the contribution of tsDMARDs to the risk of MACE, venous thromboembolism, and malignancies in certain patient populations, prompting much debate and highlighting the importance of careful risk–benefit assessment in clinical practice [291].

### 10.3. Lung Disease

Pulmonary conditions, including asthma, interstitial lung disease (ILD), and chronic obstructive pulmonary disease (COPD), are common manifestations in RA. In particular, ILD is associated with significant morbidity and mortality among patients with RA [292], and appears to be more frequent among patients with D2T disease [196,274]. A history of smoking, seropositivity, male sex, and older age are recognized as risk factors for ILD among individuals with RA [292]. Various theories have been proposed regarding the development of RA–ILD, although the exact mechanisms remain unclear [293]. In line with the mucosal origin theory, some evidence suggests the role of lungs as key mucosal sites in RA, where the influence of external triggers might lead to tolerance breakdown, resulting in an immune response against citrullinated proteins that subsequently spreads to the joint [294].

Lung involvement and anti-rheumatic drugs can interact in various ways. MTX is associated with acute pneumonitis, and it has been found to slightly increase the risk of lung disease in patients with RA compared with other treatments [295], although it may actually confer protection against the development of ILD [296]. TNF exerts pleiotropic effects on tissue remodeling [297,298], and likely contributes to pulmonary fibrosis by activating the stromal compartment and inducing the release of various growth factors (e.g., TGF-β, platelet-derived growth factor-β [PDGF-β]), cytokines (e.g., IL-4, IL-13), and chemokines [299]. Nonetheless, observational data have indicated that TNF inhibition might be linked to pulmonary adverse events and increased mortality in RA–ILD [300], prompting guidelines and practitioners to favor other treatments (e.g., abatacept or rituximab) despite low evidence levels [301,302]. However, a recent cohort study of patients with RA–ILD found no difference between TNF inhibitors and other b/tsDMARDs in mortality or respiratory hospitalization [303]. JAK/STAT signaling has also been linked to lung fibrosis, and tofacitinib-treated patients showed the lowest risk of developing ILD in a large cohort study comparing targeted therapies in RA [304].

### 10.4. Infections and Malignancies

Patients with RA are more susceptible to infections, which results from disease-related immune dysregulation, anti-rheumatic therapy leading to an immunosuppressed state, and the impact of chronic comorbidities (e.g., diabetes and lung disease) and clinical determinants. Besides, aging is linked to compromised immune surveillance, amplifying infection-related concerns among elderly patients [63]. Notably, increased rates of recurrent infections and infection-related hospital admissions have been reported among patients with D2T RA [196,305]. Various alterations of the innate and adaptive immune system have been observed in RA that are thought to contribute to infection risk, including impaired first-line defense mechanisms and a constricted T cell receptor repertoire [306]. The effect of systemic inflammation is supported by observational data that higher disease activity is associated with an elevated incidence of infections [307,308], which also implies that adequate disease control could plausibly mitigate infection risk. Nonetheless, treatment can also lead to infection. GCs are established risk factors for serious infections in RA [306], and a meta-analysis found an increase in serious infections with biological treatment in comparison with conventional DMARDs [309]. The degree to which JAK inhibitors contribute to infection risk remains uncertain [291].

Excessive and sustained inflammation, often present in D2T RA, promotes tumor growth, and individuals with RA carry a marginal but significant increase in malignancy risk compared with the general population, highlighting the need for integrating cancer screening protocols into clinical practice [310,311]. In patients with a history of cancer or in the presence of risk factors, concerns persist regarding the safety of targeted therapies, particularly JAK inhibitors. In turn, this can add complexity to therapeutic decisions in the D2T population, potentially leading to the suboptimal control of inflammation. Recommendations emphasize a risk–benefit approach that considers both the dangers of RA undertreatment and the malignancy risk of DMARDs [312].

## 11. Aging

Due to increasing life expectancy, the number of elderly individuals living with RA is growing worldwide [313], which presents a need to consider aging-related aspects in disease care and research. Furthermore, RA that develops after 60 years of age—i.e., elderly-onset RA—represents a distinct phenotype associated with a more balanced sex distribution, more acute symptom onset, higher rate of erosive disease, and more pronounced systemic involvement [314].

In the elderly population, the biological alterations associated with aging can compound the immune dysregulation in RA [63]. Aging is linked to a characteristic remodeling of the innate and adaptive immune system—i.e., immunosenescence—leading to impaired functions of T and B cells, metabolic changes, and epigenetic modifications. For instance, a specific PD-1^+^CD38^+^ subset of CX3CR1^+^ age-associated cytotoxic CD4^+^ T cells was found to be characteristically elevated in patients with D2T RA [35], implying a role in multidrug resistance. The presence of ABC subsets in the peripheral blood and synovial tissue of RA patients exemplifies B cell dysfunction in this context [122]. On the other hand, the phenomenon of inflammaging, corresponding to a sterile low-grade inflammatory state characteristic of elderly individuals, can amplify the chronic inflammation in RA [63]. The presence of CHIP, which is closely associated with elderly individuals, may impact RA pathogenesis through its influence on epigenetic programming [64], implying a further potential connection between aging and D2T disease.

Besides these immunological aspects, advancing age is characterized by increased comorbidity burden, polypharmacy, and frailty, hampering adequate use of targeted treatments [315]. Altered pharmacokinetics and narrower therapeutic windows can also complicate the pharmacological management of these patients [63]. In an elderly RA cohort treated with b/tsDMARDs, 10.5% were classified as having D2T disease, and the proportion of discontinuation due to inefficacy was significantly higher in the D2T group [316]. The complex interplay between aging and D2T RA have been examined by recent reviews in greater detail [63,317].

## 12. Treatment-Related Factors

### 12.1. Immunogenicity

Biologics have immunogenic properties, i.e., they can provoke an immune response against themselves, leading to the production of ADAs. These can neutralize therapeutic agents by blocking their antigen-binding site or promote their clearance via the formation of ICs or binding to Fcγ receptors [318], which can result in reduced clinical efficacy, especially in the context of secondary loss of response. It has been suggested that ADAs account for therapeutic non-response in a group of patients with refractory RA [9,31]. Furthermore, ADA formation may also be responsible for certain adverse events after biological therapy, including infusion reactions [318].

Concomitant use of conventional drugs, particularly MTX, attenuates the production of ADAs [319]. The clinical utility and cost-effectiveness of therapeutic drug monitoring in steering therapeutic decisions is still surrounded by uncertainty, and clinical studies evaluating switching strategies in the context of ADA formation have led to heterogeneous findings [320]. However, recent recommendations by EULAR highlight the possible benefits of a reactive monitoring strategy, whereby the measurement of biological drug levels and ADAs can help to identify a reason behind poor clinical response [321], which might be a useful instrument in D2T RA. In turn, JAK inhibitors lack immunogenicity, shifting the focus towards alternative mechanisms of non-response to tsDMARDs.

### 12.2. Adverse Events

The risk of adverse events precludes the use of certain therapeutic options in some patients, complicating the management of D2T RA. Besides, even with thorough risk–benefit assessment, drug intolerance can lead to treatment failure, worsen disease outcomes, and promote comorbidities. Safety concerns may also interfere with the T2T strategy and lead to therapeutic inertia, which is particularly relevant to the D2T concept [322]. The presence of adverse events was identified as the leading barrier to adherence in patients with either D2T or non-D2T RA [323].

Increasing concerns regarding the adverse event profile of GCs are narrowing the space for their use in RA [324]. The EULAR definition recognizes the inability to taper GC treatment as a D2T trait, and this has been confirmed by evidence from real-world cohorts reporting a higher use of GCs among patients with D2T RA [86]. The escalation of GC dose was found to be an independent risk factor for mortality in D2T disease [257]. Various adverse events also occur with DMARDs, as discussed in previous sections. In a large multi-registry study of first-line targeted therapies, TNF inhibitors and JAK inhibitors showed similar rates of discontinuation due to adverse events, while non-TNF inhibitor biologics exhibited higher rates than JAK inhibitors [325]. The use of oral GCs was associated with adverse event-related discontinuation of targeted treatments in a D2T cohort [326].

The D2T framework covers patients in whom drug intolerance or adverse events are the principal trigger of therapeutic failure. It can be postulated that the resulting D2T state in such cases may be driven by biological mechanisms that are distinct from those underlying (pre-existing or acquired) treatment resistance. Accordingly, it has been proposed that these two subtypes be considered as separate entities when investigating drivers of D2T RA [327].

## 13. Limitations

This review has several limitations. In line with the narrative design, no systematic search was performed, and the included articles were not formally appraised for quality, which may introduce selection bias. Despite the comprehensive literature search in multiple databases, some relevant studies may have been omitted, and the findings may be influenced by publication bias. The variability of study designs across the included articles further limits the ability to draw firm conclusions.

In the review, we discuss a broad range of findings on biological factors that may be relevant in the pathogenesis of D2T RA. Given the recent introduction of the D2T concept in the field of RA care and research, there is limited mechanistic evidence linking these factors to the development and perpetuation of D2T disease. Therefore, we also included findings on the determinants of treatment response derived from broader RA populations, chiefly focusing on the failure of targeted therapies. Furthermore, we summarize the underlying mechanisms of clinical characteristics (e.g., smoking, persistent pain, comorbidities) that have been linked to D2T RA in observational cohorts, even in the absence of subgroup-specific experimental data. The extent to which the pathways described in these studies operate after repeated b/tsDMARD failures remains unclear, restricting the extrapolation of existing evidence from broader cohorts or earlier disease stages and emphasizing the need for further studies conducted in the D2T population.

The EULAR definition provides a framework for the consistent identification of patients with D2T RA in clinical practice; however, its wording may give rise to ambiguity in various research applications. In the last few years, studies employing the new terminology have produced different interpretations, while others have not described their operationalization of the criteria, limiting reproducibility and comparability among D2T cohorts.

## 14. Conclusions

The emergence of the D2T framework represents a major frontier in rheumatology. As detailed in this review, the mechanisms potentially underlying D2T disease in RA are characterized by immense heterogeneity, spanning a broad spectrum of biological and clinical drivers. While some patients remain symptomatic due to persistent, multidrug-resistant synovial inflammation likely driven by the action of specific immune and stromal cell populations, others transition towards a D2T state that is chiefly governed by non-inflammatory mechanisms and/or the presence of comorbidities. Current evidence suggests that a ‘one-size-fits-all’ approach is insufficient for this population.

Recent advances using high-resolution multi-omics profiling—such as synovial transcriptomics and peripheral immunophenotyping—and modern bioinformatic approaches represent an important step towards establishing a more precise molecular taxonomy of treatment resistance. Moving forward, the clinical goal is to shift from reactive trial-and-error prescribing to a proactive stratification strategy. Through the identification of specific molecular and cellular signatures unique to each individual and their integration with clinical characteristics, clinicians may eventually be able to predict the D2T trajectory early, allowing for personalized interventions that target the true drivers of disease persistence.

## Figures and Tables

**Table 2:** Summary of studies on RA pathogenesis that specifically report on patient groups meeting the D2T RA criteria.

Ref.	Sample/ Technique	Participants	Key Findings
Akiyama et al., 2025 [35]	PB	*n* = 12 D2T RA *n* = 19 non-D2T RA *n* = 16 HCs	CX3CR1^+^CD4^+^ T cells, a cytotoxic age-associated cell subset, are elevated in D2T RA compared with non-D2T RA and HCs. The expression levels of PD-1 and CD38 by CX3CR1^+^CD4^+^ T cells are higher in D2T vs. non-D2T RA.
Gunkl-Tóth et al., 2025 (conference abstracts) [36,37]	PB	*n* = 31 D2T RA *n* = 18 non-D2T RA *n* = 32 HCs	PBMC transcriptomic analysis identified 87 DEGs (e.g., *NRG1*, *NEGR1*, *S100B*) in D2T vs. non-D2T RA. Plasma metabolomics indicated differences in amino acid and sphingolipid metabolism. Functional analyses of transcriptomic and metabolomic profiles support the involvement of neuroinflammation and neuroplasticity in D2T RA.
	fMRI		Patients with D2T RA exhibit altered connectivity and activity patterns in the somatosensory area and the PCC. After acute pain stimulation, the D2T group showed differential alterations in connectivity patterns compared with non-D2T patients and HCs.
Nishiura et al., 2025 (conference abstract) [38]	PB	*n* = 13 D2T RA *n* = 13 non-D2T RA	PB transcriptomic analysis revealed 2875 DEGs between matched D2T and non-D2T RA samples. Functional enrichment analysis showed increased expression of genes related to T-cell-mediated immunity, innate immunity, and TLR signaling (especially the TLR7 pathway) in the D2T group.
Yan et al., 2025 [39]	PB	*n* = 325 D2T RA *n* = 239 treatment-naïve RA *n* = 478 RA in remission under b/tsDMARDs *n* = 339 HCs	PB counts of total T cells, CD4^+^ T, and T_reg_ cells are lower in D2T RA than in all other groups. T_H17_/T_reg_ ratio is increased in both D2T RA and remission RA compared with treatment-naïve RA and HCs. Treatment with low-dose IL-2 increases the number of B cells, T cells, and T cell subsets, and decreases T_H17_/T_reg_ ratio across all RA groups.
MacDonald et al., 2024 [40]	SB	*n* = 18 active RA (including D2T RA) *n* = 9 RA in sustained remission *n* = 7 HCs	AXL^+^ synovial tissue DC2s are decreased in active RA, especially in the D2T subgroup. The proportion of classical CXCL13^+^ T_PH_ cells is highest in D2T RA, and significantly elevated compared with the remission group.
Giollo et al., 2025 [41]	SB	*n* = 43 PIRRA (including *n* = 19 D2T/PIRRA) *n* = 21 NIRRA (including *n* = 10 D2T/NIRRA)	Patients with NIRRA are more likely to have a fibroid/pauci-immune synovial pathotype compared with the PIRRA group, with an even stronger association in D2T RA.
Ruiz-Limón et al., 2025 [42]	Stool	*n* = 13 D2T RA *n* = 26 stable RA under csDMARD monotherapy	The phylum Bacteroidetes and the genus *Megasphaera* is enriched in D2T RA, whereas the families Lachnospiraceae and Pasteurellaceae (and the respective genera *Coprococcus* and *Haemophilus*) are more abundant in stable RA. A higher Firmicutes/Bacteroidetes ratio is associated with a decreased risk of D2T RA.

Abbreviations: b, biological; D2T, difficult-to-treat; DC2, type 2 dendritic cell; DEG, differentially expressed gene; DMARD, disease-modifying anti-rheumatic drug; fMRI, functional magnetic resonance imaging, HC, healthy control; NIRRA, non-inflammatory refractory rheumatoid arthritis; PIRRA, persistent inflammatory refractory rheumatoid arthritis; PB, peripheral blood; PBMC, peripheral blood mononuclear cell; PCC, posterior cingulate cortex; PD-1, programmed cell death protein 1; RA, rheumatoid arthritis; SB, synovial biopsy; TLR, Toll-like receptor; T_reg_, regulatory T cell; ts, targeted synthetic.

## Data Availability

No new data were created or analyzed in this study. Data sharing is not applicable to this article.

## Abbreviations

The following abbreviations are used in this manuscript:

α7nAChR	α7 nicotinic acetylcholine receptor
ABC	Age-associated B cell
ACPA	Anti-citrullinated protein antibody
ACR20	American College of Rheumatology 20% response
ADA	Anti-drug antibody
AKT	Protein kinase B
anti-CarP	Anti-carbamylated protein antibody
APC	Antigen-presenting cell
b	Biological
BCMA	B-cell maturation antigen
BDNF	Brain-derived neurotrophic factor
BiTE	Bispecific T cell engager
BMI	Body mass index
CAD	Coronary artery disease
CAR	Chimeric antigen receptor
CDK	Cyclin-dependent kinase
CGRP	Calcitonin gene-related peptide
CHIP	Clonal hematopoiesis of indeterminate potential
CII	Collagen type II
CNS	Central nervous system
COMP	Cartilage oligomeric matrix protein
COPD	Chronic obstructive pulmonary disease
CRP	C-reactive protein
cs	Conventional synthetic
CTAP	Cell-type abundance phenotype
CVD	Cardiovascular disease
D2T	Difficult-to-treat
DAS	Disease Activity Score
DC	Dendritic cell
DC2	Type 2 dendritic cell
DEG	Differentially expressed gene
DMARD	Disease-modifying anti-rheumatic drug
DMN	Default mode network
DNA	Deoxyribonucleic acid
DRG	Dorsal root ganglia
EPC	Endothelial progenitor cell
EULAR	European Alliance of Associations for Rheumatology
FAP	Fibroblast activation protein
FLS	Fibroblast-like synoviocyte
FM	Fibromyalgia
fMRI	Functional magnetic resonance imaging
Fc	Fragment crystallizable
GC	Glucocorticoid
GM-CSF	Granulocyte-macrophage colony-stimulating factor
GWAS	Genome-wide association study
HbA1c	Hemoglobin A1c
HC	Healthy control
HDL	High-density lipoprotein
HIF-1α	Hypoxia-inducible factor 1 alpha
HLA	Human leukocyte antigen
IC	Immune complex
IFN	Interferon
IgG	Immunoglobulin G
IL	Interleukin
ILD	Interstitial lung disease
JAK	Janus kinase
LDL	Low-density lipoprotein
MACE	Major adverse cardiovascular events
MAPK	Mitogen-activated protein kinase
mDC	Myeloid dendritic cell
miRNA	MicroRNA
MMP	Matrix metalloproteinase
mPFC	Medial prefrontal cortex
mTOR	Mechanistic target of rapamycin
MTX	Methotrexate
NET	Neutrophil extracellular trap
NF-κB	Nuclear factor kappa B
NIRRA	Non-inflammatory refractory rheumatoid arthritis
NLR	Neutrophil-to-lymphocyte ratio
NLRP3	NLR family pyrin domain containing 3
NMDA	N-methyl-D-aspartate
PB	Peripheral blood
PBMC	Peripheral blood mononuclear cell
PCC	Posterior cingulate cortex
PD-1	Programmed cell death protein 1
PDGF-β	Platelet-derived growth factor-β
PGE_2_	Prostaglandin E_2_
PhGA	Physician global assessment
PI3K	Phosphoinositide 3-kinase
PIRRA	Persistent inflammatory refractory rheumatoid arthritis
PPAD	*Porphyromonas gingivalis* peptidylarginine deiminase
pre-DC	Dendritic cell precursor
RA	Rheumatoid arthritis
RDCI	Rheumatic disease comorbidity index
RF	Rheumatoid factor
RNA	Ribonucleic acid
RNA-Seq	RNA sequencing
ROS	Reactive oxygen species
SB	Synovial biopsy
SCFA	Short-chain fatty acid
SE	Shared epitope
SGK1	Serum/glucocorticoid-regulated kinase 1
sICAM1	Soluble intercellular adhesion molecule 1
SIRT	Sirtuin
SNP	Single-nucleotide polymorphism
STAT	Signal transducer and activator of transcription
sTNFR	Soluble tumor necrosis factor receptor
T2D	Type 2 diabetes
T2T	Treat-to-target
T_ang_	Angiogenic T cell
T_FH_	Follicular helper T cell
TGF-β	Transforming growth factor-β
TLR	Toll-like receptor
TNF	Tumor necrosis factor
T_PH_	Peripheral helper T cell
T_reg_	Regulatory T cell
ts	Targeted synthetic
VNS	Vagus nerve stimulation
